# The Yield of Staging Investigations in Patients with Breast Cancer Planned for Neoadjuvant Chemotherapy

**DOI:** 10.3390/curroncol33040203

**Published:** 2026-04-01

**Authors:** Kadhim Taqi, Camelia Ursu, Susan Isherwood, Rabia Raheel, Julia Downey, Jeffrey Q. Cao, Sasha Lupichuk, Omar Khan, Nancy Nixon, May Lynn Quan, Alison Laws

**Affiliations:** 1 Division of Surgical Oncology, Department of Surgery, University of Calgary, Calgary, AB T2N 1N4, Canada; 2 Division of Surgical Oncology, Department of Surgery, Sultan Qaboos Comprehensive Cancer Care and Research Center, University Medical City, Muscat 123, Oman; 3 Cumming School of Medicine, University of Calgary, Calgary, AB T2N 1N4, Canada; 4 Department of Surgery, University of Calgary, Calgary, AB T2N 1N4, Canada; 5 Department of Community Health Sciences, University of Calgary, Calgary, AB T2N 1N4, Canada; 6 Division of Radiation Oncology, Arthur J.E. Child Comprehensive Cancer Centre, Calgary, AB T2N 1N4, Canada; 7 Department of Oncology, University of Calgary, Calgary, AB T2N 1N4, Canada; 8 Division of Medical Oncology, Department of Oncology, University of Calgary, Calgary, AB T2N 1N4, Canada

**Keywords:** staging investigations, breast cancer, neoadjuvant chemotherapy, locally advanced, systemic staging

## Abstract

Background: Patients with breast cancer planned for neoadjuvant chemotherapy (NAC) represent a diverse population including high-risk early breast cancer (EBC) and locally advanced breast cancer (LABC). Staging investigations are routinely performed, but the clinical utility is unclear. Methods: Using a provincial cancer registry, we identified breast cancer patients referred for NAC between 2020 and 2022. Patients with staging investigations were stratified into EBC (anatomic clinical stage I–II) and LABC (stage III). Rates of metastatic (M1) disease and associated factors were assessed. Results: Among 529 EBC patients, 515 (97.4%) underwent staging. The M1 disease rate was 5.4%. The M1 rate for cT1-2N0 was 1.1%, and for cT1-2N1 it was 7.9%. In multivariable analysis, cT1N1 (OR 5.31; 95% CI 1.05–27.0; *p* = 0.044) and cT2N1 (OR 4.59; 95% CI 1.02–20.67; *p* = 0.047) were associated with M1 disease in EBC. All 320 LABC patients underwent staging. The M1 disease rate was 22.8%, significantly higher than in EBC (*p* < 0.001). A higher cT/N stage correlated with M1 disease in LABC, although most subgroups demonstrated rates of ≥10%. Conclusions: These findings support a risk-adapted approach to pre-treatment staging in patients planned for NAC, omitting staging in asymptomatic cT1–2N0 disease, considering it for node-positive EBC, and performing it routinely in LABC.

## Simple Summary

Radiological imaging is commonly used in patients with breast cancer to assess disease stage before starting chemotherapy, but its benefit varies by stage. Using population-wide data, we studied how often these tests detected cancer that had already spread in patients planned for chemotherapy before surgery. We found that cancer spread was very uncommon in patients with small tumors and no lymph node involvement, even when chemotherapy was planned, suggesting that routine staging scans can be safely avoided in this group. In contrast, patients with larger tumors or lymph node involvement had a higher likelihood of cancer spread. These results support more selective use of staging tests, which could reduce unnecessary scans, costs, patient anxiety, and treatment delays, while helping refine future guidelines and policy decisions.

## 1. Introduction

Staging investigations alter the management of patients newly diagnosed with breast cancer when distant metastatic disease is identified. In patients with locoregionally advanced breast cancer (LABC), systemic staging is recommended due to the relatively high likelihood of detecting distant metastases [1,2,3]. Conversely, the diagnostic yield of staging is much lower for early breast cancer (EBC), and thus its use is more debated, with current guidelines varying in their recommendations. The National Comprehensive Cancer Network (NCCN), American Society of Clinical Oncology (ASCO) and Cancer Care Ontario discourage routine staging for asymptomatic patients with stage I or II disease [1,4,5]. In contrast, the European Society for Medical Oncology (ESMO) supports the use of staging for patients with node-positive disease, tumors larger than 5 cm (T3), or aggressive tumor biology (3), while the American College of Radiology (ACR) recommends staging for patients with stage IIB or higher disease [6].

Patients referred for neoadjuvant chemotherapy (NAC) represent a distinct subgroup encompassing both early and locally advanced disease. These patients often present with higher-risk features that render them eligible for NAC such as node positivity or more aggressive biologic subtypes (even when presenting with a lower anatomic clinical stage). The diagnostic yield and clinical utility of staging investigations in this population is not well-characterized. Multiple studies have shown high rates of staging in patients with EBC, especially before NAC initiation [7,8,9]. Overuse of staging investigations is costly [7,10] and may expose patients to harms such as unnecessary radiation, invasive follow-up procedures, and anxiety over false-positive findings [11]. Further, staging workups can delay the timely initiation of therapy, potentially impacting treatment outcomes [12]. Nevertheless, accurate assessment of disease extent remains crucial for prognostication and to guide treatment strategies, including avoidance of chemotherapy toxicity for some patients when metastatic disease is confirmed.

Across the six tertiary and regional cancer centers in the province of Alberta, Canada, distant staging investigations are performed routinely for patients with breast cancer planned for NAC. The objective of this study was to evaluate the rate of de novo metastatic (M1) disease detected in staging investigations among patients referred for NAC, stratified by EBC and LABC, and to identify factors associated with M1 disease.

## 2. Materials and Methods

Institutional review board approval was obtained from the Health Research Ethics Board of Alberta–Cancer Committee (HREBA-CC), and a waiver of consent was granted given the minimal-risk nature of the study. All patients with breast cancer referred to medical oncology for consideration of NAC between 2020 and 2022 were identified from the Alberta Cancer Registry, which captures all new cancers in the province of approximately five million individuals. Patients who did not undergo any distant staging investigations were excluded. Two cohorts were generated based on the clinical T/N stage (cT/cN) at presentation according to the American Joint Committee on Cancer (AJCC) 8th edition anatomic staging system. The EBC cohort was defined as stage I-II (cT1-2 N0-1 or cT3N0) and the LABC cohort as stage III (cT3N1, any cT4, and any cN2-3).

The primary outcome of interest was final metastatic status after staging (M0 vs. M1), obtained from the Alberta Cancer Registry and confirmed in the medical record. Details of the staging workup were obtained from medical chart review. Each distant staging investigation was documented, including computed tomography (CT) of the chest and abdomen with or without pelvis (CA ± P), bone scan, brain imaging (CT or Magnetic Resonant Imaging (MRI)) and positron emission tomography (PET). Staging results according to the reporting radiologist’s interpretation were categorized as negative (any findings considered likely benign), indeterminate (findings could not be confidently classified as benign or malignant) or suspicious (findings were concerning for malignancy and considered likely metastatic). Further workup was defined as additional imaging tests or biopsies prompted by findings on the baseline staging scans.

Remaining demographic, disease and treatment characteristics were obtained from medical chart review. Histological type, grade, estrogen receptor (ER), progesterone receptor (PR), and human epidermal growth factor receptor-2 (HER2) status were obtained from the pre-treatment core biopsy. Hormone receptor positivity (HR+) and HER2 positivity (HER2+) were defined according to the American Society of Clinical Oncology and the College of American Pathologists (ASCO/CAP) guidelines [13] and were used to construct biologic subtype categories of HR+/HER2−, HER2+ and triple-negative (TN).

### Statistical Analysis

The EBC and LABC cohorts were analyzed separately. Descriptive statistics were used to summarize demographic and disease characteristics, with continuous variables reported as medians with interquartile ranges (IQRs) and categorical variables as frequencies and proportions. Time from pathologic diagnosis (biopsy date) to NAC initiation was compared by staging result using Mann–Whitney U tests. Associations between clinical factors (age, cT and cN, histologic type, grade, and biologic subtype) and M1 status were evaluated using univariable logistic regression. Factors with statistically significant associations were included in multivariable logistic regression models. For the EBC cohort, the multivariable model included cT/N category, grade and biologic subtype (histologic type was excluded due to a small sample size of lobular cancers). For the LABC cohort, samples sizes were small for individual cT/N categories. As such, two models were generated, one including cT and cN as separate variables and one using AJCC subgroups of stage III (A vs. B vs. C), along with age and grade. For both cohorts, we reported absolute rates of M1 disease for pre-planned subgroups of cT/N category and biologic subtype, reflecting groups for which staging investigations have been recommended in some guidelines. All analyses were performed using StataIC version 16.1 (StataCorp LLC, College Station, TX, USA). A *p*-value of <0.05 was considered statistically significant.

## 3. Results

### 3.1. Early Breast Cancer Cohort (Stage IA-IIB)

**Figure 1:**
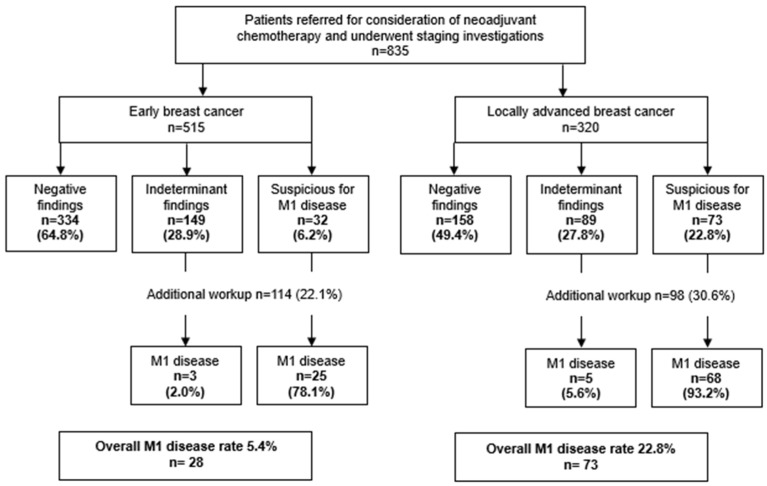
Results of staging investigations in patients with early and locoregionally advanced breast cancer referred for neoadjuvant chemotherapy.

Among 529 eligible patients with EBC, 14 patients had no staging investigations and were excluded. The remaining 515 (97.4%) underwent CT CA +/− P and/or bone scan and were included for analysis. Most patients (*n* = 469, 91.1%) had guideline-concordant staging with both CT CA ± P and bone scan. Additional brain imaging was performed for 48 patients (9.3%) and an additional PET scan for 4 patients (0.8%). Detailed staging results are shown in Figure 1. Overall, M1 disease was identified in 28/515 patients (5.4%; 95% CI, 3.8–7.7%). Sites of distant metastases were as follows: lung (*n* = 14, 50.0%), bone (*n* = 12, 42.9%), liver (*n* = 11, 39.3%), brain (*n* = 1, 3.6%), and other (*n* = 9, 32.2%). Among M0 patients, those who required additional workup (*n* = 114, 22.1%) after baseline staging scans experienced a median delay of five days to NAC initiation (43 vs. 38 days; *p* = 0.03).

Clinical characteristics stratified by M0 versus M1 disease are presented in Table 1. Clinical T/N stage, histology, grade and biologic subtype were all significantly associated with M1 status (all *p* < 0.05) in univariable analyses. In multivariable analysis, only the cT1N1 (OR 5.31; 95% CI 1.05–27.0; *p* = 0.044) and cT2N1 (OR 4.59; 95% CI 1.02–20.67; *p* = 0.047) stage remained significantly associated with higher odds of M1 disease (Table 2). While not statistically significant, TN or HER2+ subtypes and grade 3 tumors showed lower odds of M1 disease. Rates of M1 disease in pre-specified subgroups of cT/N category and biologic subtype are presented in Table 3. Most notably, the M1 rate was 1.1% (2/174) for cT1-2N0, 5.4% (2/37) for cT3N0 and 7.9% (24/302) for cT1-2N1.

### 3.2. Locally Advanced Breast Cancer Cohort (Stage III)

Among 320 eligible patients with LABC, all (100%) underwent CT CA +/− P and/or bone scan and were included for analysis. Most patients (*n* = 293; 91.5%) received guideline-concordant staging with both CT CA ± P and a bone scan. Additional brain imaging was performed in 40 patients (12.5%) and an additional PET scan in 7 patients (2.2%). Detailed staging results are shown in Figure 1. Overall, M1 disease was identified in 73/320 patients (22.8%; 95% CI, 18.6–27.7%), a significantly higher rate than the EBC cohort (*p* < 0.001). Sites of distant metastases were as follows: bone (*n* = 41, 56.2%), lung (*n* = 39, 53.4%), liver (*n* = 29, 39.7%), brain (*n* = 3, 4.1%) and other (*n* = 25, 34.2%). Among M0 patients, those who required additional workup after baseline staging scans did not experience any delay to NAC initiation (median 36 days for both, *p* = 0.54).

Clinical characteristics stratified by M0 versus M1 disease are shown in Table 4. Age, cT and cN categories as well as overall AJCC stage, and grade were significantly associated with M1 disease (all *p* < 0.05) in univariable analyses. In multivariable analysis, cT4 and cN3 disease remained significantly associated with higher odds of M1 disease, whereas stage IIIA disease (vs. IIIB or IIIC) and grade 3 were associated with significantly lower odds of M1 (all *p* < 0.05, Supplementary Tables S1 and S2). Notably, all cT, cN, and grade subgroups for the LABC cohort had absolute M1 rates ≥ 10%. The absolute rate of M1 disease for stage IIIA was 9.2% (12/130); rates for each T/N category are shown in Table 3.

## 4. Discussion

This study uniquely uses a large population-based cohort of breast cancer patients referred for NAC with unselected use of staging investigations, allowing for an accurate and generalizable assessment of the diagnostic yield of staging in this population. We found a low rate of M1 disease among patients with cT1–T2N0 disease (1% overall), supporting the omission of routine staging in this population even when NAC is planned, which is consistent with current guidelines for all-comers with early breast cancer [3,4,5]. In contrast, the incidence of M1 disease was higher for EBC patients with cT3N0 (5%) and N1 (8–9%) disease, suggesting that staging may be justified in these subgroups. Notably, a trend toward higher M1 rates was also observed among patients with ER+/HER2− subtype and grade 1–2 tumors. While these did not reach statistical significance in multivariable analysis, the low event rate may have limited power. Nonetheless, our findings do not clearly support preferential use of staging in aggressive subtypes (e.g., TN and HER2+) even at a lower clinical stage of disease, as some guidelines have suggested, though this observation should be interpreted with caution. In contrast, patients with LABC exhibited a significantly higher burden of metastatic disease, with even the lowest-risk subgroups demonstrating M1 rates of ≥9–10%, reinforcing guideline recommendations for routine staging in this population.

Most studies evaluating the diagnostic yield of staging investigations in breast cancer included all-comers and were stratified by stage. In such studies, the overall rate of M1 disease is low (0–6%) for EBC populations [11,13,14] but significant for LABC (18–36%) [8,10,15,16,17]. The likelihood of M1 disease has also been generally consistent across tumor subtypes [13,14]. Cohorts of high-risk subgroups such as patients referred for NAC are more limited. A retrospective review from Memorial Sloan Kettering Cancer Centre [7] included 303 NAC patients with stage I-II disease, of whom 85% had staging investigations, mostly with PET/CT. The M1 rate detected by staging was 4.9%, almost all (*n* = 14/15) in patients with cN1 disease. They concluded that pre-NAC staging is not necessary for patients with EBC. A study from the United Kingdom evaluated 163 patients referred for NAC where staging was routine. The overall rate of M1 disease was 9.3%, but in the cohort with T1-3N0, the rate was only 1.6% [9]. Our findings are aligned with these prior studies, but our larger sample size allowed for further stratification by stage and biologic subtype to develop recommendations more tailored to individual tumor characteristics.

In our cohort, staging investigations resulted in small delays to treatment (median 5 days) for patients who required additional workup after baseline scans. While time-to-treatment benchmarks are lacking for NAC patients specifically, delays of ≥4–8 weeks from breast cancer diagnosis to treatment are associated with worse outcomes in those undergoing surgery first [18,19,20]. As such, the differences in time to treatment observed in our study are likely clinically insignificant. Other studies have found delays of 6–14 days for patients who undergo staging [21], so this issue warrants consideration in the local context.

Several limitations of this study should be acknowledged. Despite the large overall sample of 835 patients, stratification by clinical T/N stage and biologic subtype resulted in small subgroup sizes, limiting the precision of some estimates. Similarly, multivariable analyses—particularly in the EBC cohort—may have been underpowered given the relatively low event rate. Given the retrospective nature of this study, there is a possibility of selection bias in staging use and potential for heterogeneity in the type of modality chosen, both of which could influence diagnostic yield. However, exclusions due to non-performance of staging were very minimal (*n* = 14 patients, 1.6%) and type of staging was quite homogenous, with 91% of patients in both EBC and LABC cohorts undergoing guideline-concordant CT CA +/− P and bone scan. Collectively, this supports the minimal risk of these issues. PET scan was infrequently used in this population, limiting the generalizability of our findings to centers where this modality is routinely incorporated. PET has been shown to increase the detection of M1 disease and is being increasingly incorporated into clinical practice guidelines, primarily for LABC [1,4,22]. In addition, clinical staging was used for group classification, which may be subject to misclassification, particularly with respect to nodal assessment. However, this reflects the reality of available information before NAC is initiated. Finally, with any retrospective medical chart review, there is a risk of variable misclassification, though variables included in this study were generally clearly documented.

## 5. Conclusions

Based on our findings, we advocate for the omission of routine staging investigations for asymptomatic patients with cT1-2N0 disease planned for NAC, in keeping with current guideline recommendations. EBC patients with cT3N0 or N1 disease may be considered for staging, and staging should be routine for those with LABC. Staging guidelines in our province are currently under review and will be informed by this study, and our results are widely generalizable to other jurisdictions.

## Figures and Tables

**Table 1:** Baseline characteristics for early breast cancer (stage I–II) patients with M0 vs. M1 disease.

Variable	M0 (*n* = 487)	M1 (*n* = 28)	*p*-Value
**Median age in years (IQR)**	50 (42–60)	53 (47–62)	0.17
**cT/N stage**			0.04
T1N0	36 (7.4%)	0 (0%)	
T2N0	138 (28.3%)	2 (7.1%)	
T1N1	71 (14.6%)	7 (25.0%)	
T2N1	207 (42.5%)	17 (60.7%)	
T3N0	35 (7.2%)	2 (7.1%)	
**Histology**			0.02
Ductal	473 (97.1%)	25 (89.3%)	
Lobular	14 (2.9%)	3 (10.7%)	
**Grade**			0.04
1–2	158 (32.4%)	15 (53.6%)	
3	326 (66.9%)	13 (46.4%)	
**ER Status**			0.002
Positive	238 (48.9%)	22 (78.6%)	
Negative	249 (51.1%)	6 (21.4%)	
**PR Status**			0.015
Positive	153 (31.4%)	15 (53.6%)	
Negative	334 (68.6%)	13 (46.4%)	
**HER2 Status**			0.33
Positive	184 (37.8%)	8 (28.6%)	
Negative	303 (62.2%)	20 (71.4%)	
**Biologic subtype**			0.005
HR+/HER2−	126 (25.9%)	15 (53.6%)	
TN	177 (36.3%)	5 (17.9%)	
HER2+	184 (37.8%)	8 (28.5%)	

IQR: interquartile range. cT: clinical tumor stage. cN: clinical nodal stage. HR: hormone receptor. TN: triple-negative. HER2: human epidermal growth factor receptor-2.

**Table 2:** Multivariable analysis evaluating factors associated with M1 status in the early breast cancer (stage I–II) cohort.

Variable	Unadjusted Odds Ratio	Adjusted Odds Ratio	Adjusted 95% Confidence Interval	Adjusted *p*-Value
**cT/N stage**				
T1N0	No events	No events	-	-
T2N0	Reference	Reference		
T3N0	3.94	2.35	0.30–18.28	0.414
T1N1	6.80	5.31	1.05–26.99	0.044
T2N1	5.67	4.59	1.02–20.67	0.047
**Grade**				
1–2	Reference	Reference		
3	0.40	0.48	0.22–1.08	0.076
**Biologic subtype**				
HR+/HER2−	Reference	Reference		
TN	0.24	0.43	0.14–1.27	0.125
HER2+	0.37	0.50	0.20–1.24	0.134

cT stage: clinical tumor stage. cN stage: clinical nodal stage. HR: hormone receptor. TN: triple-negative. HER2: human epidermal growth factor receptor-2.

**Table 3:** Absolute rates of M1 disease by cT/N stage and biologic subtype.

Overall AJCC Stage	cT/N	All Biologic Subtypes	HR+/HER2−	TN	HER2+
IA	T1N0	0/36 (0%)	--	--	--
IIA	T2N0	2/138 (1.4%)	0/17 (0%)	0/69 (0%)	2/52 (3.7%)
	T1N1	7/78 (9.0%)	5/28 (17.9%)	1/25 (4.0%)	1/25 (4.0%)
IIB	T3N0	2/37 (5.4%)	2/19 (10.5%)	0/12 (0%)	0/6 (0%)
	T2N1	17/224 (7.6%)	8/75 (10.7%)	4/60 (6.7%)	5/89 (5.6%)
IIIA	T1-2N2	2/43 (4.7%)	1/17 (5.9%)	0/10 (0%)	1/16 (6.3%)
	T3N1	9/77 (11.7%)	5/36 (13.9%)	1/13 (7.7%)	3/28 (10.7%)
	T3N2	1/10 (10.0%)	*	*	*
IIIB	T4N0	4/17 (23.5%)	*	*	*
	T4N1	23/80 (28.8%)	13/42 (31.0%)	1/12 (8.3%)	9/26 (34.6%)
	T4N2	7/19 (36.8%)	*	*	*
IIIC	T1-2N3	9/24 (37.5%)	4/13 (30.8%)	1/4 (25.0%)	4/7 (57.1%)
	T3N3	4/16 (25.0%)	*	*	*
	T4N3	14/33 (42.4%)	3/7 (42.9%)	7/12 (58.3%)	4/14 (28.6%)

cT stage: clinical tumor stage. cN stage: clinical nodal stage. HR: hormone receptor. TN: triple-negative. HER2: human epidermal growth factor receptor-2. * Omitted due to small sample size (<20 patients in total).

**Table 4:** Baseline characteristics for locally advanced patients (stage III) with M0 vs. M1 disease.

Variable	M0 (*n* = 247)	M1 (*n* = 73)	*p*-Value
**Median age in years (IQR)**	49 (41–62)	57 (44–67)	0.01
**cT**			<0.001
1–2	57 (23.0%)	11 (15.0%)	
3	89 (36.0%)	14 (19.2%)	
4a-c	39 (15.8%)	30 (41.1%)	
4d	62 (25.1%)	18 (25.7%)	
**cN**			0.009
0	13 (5.3%)	4 (5.5%)	
1	125 (50.6%)	32 (43.8%)	
2	62 (25.1%)	10 (13.7%)	
3	47 (19.0%)	27 (37.0%)	
**Stage**			<0.001
IIIA (T3N1 and T0-3 N2)	118 (47.8%)	12 (16.4%)	
IIIB (T4 N0-2)	82 (33.2%)	34 (46.6%)	
IIIC (T any N3)	47 (19.0%)	27 (37.0%)	
**Histology**			0.74
Ductal	240 (97.2%)	72 (98.6%)	
Lobular	6 (2.4%)	1 (1.4%)	
**Grade**			0.003
1–2	68 (27.5%)	34 (46.6%)	
3	179 (72.5%)	39 (53.4%)	
**ER Status**			0.23
Positive	150 (60.7%)	50 (68.5%)	
Negative	97 (39.3%)	23 (31.5%)	
**PR Status**			0.33
Positive	106 (42.9%)	36 (49.3%)	
Negative	141 (57.1%)	37 (50.7%)	
**HER2 Status**			0.91
Positive	83 (33.6%)	24 (32.9%)	
Negative	164 (66.4%)	49 (67.1%)	
**Biologic subtype**			0.76
HR+/HER2−	108 (43.7%)	35 (48.0%)	
TN	56 (22.7%)	14 (19.2%)	
HER2+	83 (33.6%)	24 (32.9%)	

IQR: interquartile range. cT: clinical tumor stage. cN: clinical nodal stage. HR: hormone receptor. TN: triple-negative. HER2: human epidermal growth factor receptor-2.

## Data Availability

The datasets generated during and/or analyzed during the current study are not publicly available due to patient privacy but are available from the corresponding author on reasonable request.

## Supplementary Materials

The following supporting information can be downloaded at: https://www.mdpi.com/article/10.3390/curroncol33040203/s1, Table S1: Multivariable analysis #1 evaluating factors associated with M1 status in the locally advanced (stage III) cohort, including age, cT, cN and grade; Table S2: Multivariable analysis #2 evaluating factors associated with M1 status in the locally advanced (stage III) cohort, including age, overall AJCC stage and grade.

## Abbreviations

The following abbreviations are used in this manuscript:

LABC	Locally advanced breast cancer
EBC	Early breast cancer
NCCN	National Comprehensive Cancer Network
ASCO	American Society of Clinical Oncology
ESMO	European Society of Medical Oncology
ACR	American College of Radiology
NAC	Neoadjuvant chemotherapy
M1	De novo metastatic
AJCC	American Joint Committee on Cancer
CT	Computed tomography
MRI	Magnetic resonant imaging
PET	Positron emission tomography
CA ± P	Chest and abdomen with or without pelvis
ER	Estrogen receptor
PR	Progesterone receptor
HER2	Epidermal growth factor receptor-2
HR+	Hormone receptor positivity
HER2+	HER2 receptor positivity
CAP	College of American Pathologists
IQRs	Interquartile ranges
TN	Triple-negative
OR	Odds ratio

## References

[1] Gradishar W., Moran M., Abraham J., Abramson V., Aft R., Agnese D., Allison K., Anderson B., Bailey J., Burstein H. (2025). National Comprehensive Cancer Network. NCCN Clinical Practice Guidelines in Oncology: Breast Cancer.

[2] American Society of Clinical Oncology, American Board of Internal Medicine Choosing Wisely: Cancer Tests and Treatments. Published 2012. http://www.choosingwisely.org/patient-resources/cancer-tests-and-treatments.

[3] Loibl S., André F., Bachelot T., Barrios C., Burstein H., Cadoso M., Carey L., Dawood S., Del Mastro L., Denkert C. (2024). Early breast cancer: ESMO Clinical Practice Guideline for diagnosis, treatment and follow-up. Ann. Oncol..

[4] Arnaout A., Varela N.P., Allarakhia M., Grimard L., Hey A., Lau J., Thain L., Eisen A. (2020). Baseline staging imaging for distant metastasis in women with stage I, II, and III breast cancer. Curr. Oncol..

[5] American Society of Clinical Oncology Choosing Wisely. ASCO. https://www.asco.org/news-initiatives/current-initiatives/cancer-care-initiatives/value-cancer-care/choosing-wisely.

[6] McDonald E.S., Scheel J.R., Lewin A.A., Weinstein S., Dodelzon K., Dogan B., Fitzpatrick A., Kuzmiak C., Newell M., Expert Panel on Breast Imaging (2024). ACR Appropriateness Criteria^®^ Imaging of Invasive Breast Cancer. J. Am. Coll. Radiol..

[7] Srour M.K., Lee M., Walcott-Sapp S., Luu M., Chung A., Giuliano A., Amersi F. (2019). Overuse of preoperative staging of patients undergoing neoadjuvant chemotherapy for breast cancer. Ann. Surg. Oncol..

[8] Roszkowski N., Lam S.S., Copson E., Cutress R.I., Oeppen R. (2021). Expanded criteria for pretreatment staging CT in breast cancer. BJS Open.

[9] Broadbent R., Ralston S., Lauder J., Howell S. (2020). Outcome of CT staging prior to neoadjuvant chemotherapy in patients with early breast cancer. Breast J..

[10] Srour M.K., Lee M., Walcott-Sapp S., Luu M., Chung A., Giuliano A., Amersi F. (2021). Incidental radiologic findings in breast cancer patients who undergo staging prior to neoadjuvant chemotherapy. Breast J..

[11] Kamel D., Youssef V., Hopman W.M., Mates M. (2021). Staging investigations in asymptomatic early breast cancer patients at the Cancer Centre of Southeastern Ontario. Curr. Oncol..

[12] Lohrisch C., Paltiel C., Gelmon K., Speers C., Taylor S., Barnett J., Olivotto I. (2006). Impact on survival of time from definitive surgery to initiation of adjuvant chemotherapy for early-stage breast cancer. J. Clin. Oncol..

[13] Allison K.H., Hammond M.E.H., Dowsett M., McKernin S., Carey L., Fitzgibbons P., Hayes D., Lakhani S., Chavez-MacGregor M., Perlmutter J. (2020). Estrogen and progesterone receptor testing in breast cancer: ASCO/CAP guideline update. J. Clin. Oncol..

[14] Bychkovsky B.L., Guo H., Sutton J., Spring L., Faig J., Dagogo-Jack I., Battelli C., Houlihan M.J., Yeh T.C., Come S.E. (2016). Use and Yield of Baseline Imaging and Laboratory Testing in Stage II Breast Cancer. Oncologist.

[15] Barrett T., Bowden D.J., Greenberg D.C., Brown C., Wishart G., Britton P. (2009). Radiological staging in breast cancer: Which asymptomatic patients to image and how. Br. J. Cancer.

[16] Alkazaz A.A., Ali N.F., Salman A.Z., Almahari S., Altaei T., Albati W., Habib H., Alsadoon A., Almawlani N., Alkhabbaz F. (2024). Need for staging investigations in newly diagnosed breast cancer: Establishing local guidelines for radiological staging in Bahrain. Eur. J. Breast Health.

[17] Ali B., Mubarik F., Zahid N., Sattar A.K. (2020). Clinicopathologic features predictive of distant metastasis in patients diagnosed with invasive breast cancer. JCO Glob. Oncol..

[18] Luz F.A.C.D., Marinho E.D.C., Nascimento C.P., Marques L.D.A., Delfino P.F.R., Antonioli R.M., de Araujo R.A., Silva M.J.B. (2022). The effect of delayed treatment in breast cancer patients: How much is too late? An experience of a single-center study effect of surgery delay in survival. Surg. Oncol..

[19] Wiener A.A., Hanlon B.M., Schumacher J.R., Vande Walle K.A., Wilke L.G., Neuman H.B. (2023). Reexamining time from breast cancer diagnosis to primary breast surgery. JAMA Surg..

[20] Bleicher R.J., Ruth K., Sigurdson E.R., Beck J.R., Ross E., Wong Y., Patel S., Boraas M., Chang E., Topham N. (2016). Time to surgery and breast cancer survival in the United States. JAMA Oncol..

[21] Bleicher R.J., Ruth K., Sigurdson E.R., Ross E., Wong Y., Patel S., Boraas M., Topham N., Egleston B. (2012). Preoperative delays in the US Medicare population with breast cancer. J. Clin. Oncol..

[22] Vaz S.C., Woll J.P.P., Cardoso F., Groheux D., Cook G., Ulaner G., Jacene H., Rubio I., Schoones J., Peeters M.-J. (2024). Joint EANM-SNMMI guideline on the role of 2-[^18^F]FDG PET/CT in no special type breast cancer (endorsed by the ACR, ESSO, ESTRO, EUSOBI/ESR, and EUSOMA). Eur. J. Nucl. Med. Mol. Imaging.

