# Supplementary material for: The Yield of Staging Investigations in Patients with Breast Cancer Planned for Neoadjuvant Chemotherapy
Source: Curr Oncol. 2026 Apr 1;33(4):203. doi: 10.3390/curroncol33040203 (PMC13114693; doi:10.3390/curroncol33040203)
Supplement: Supplementary file 1 [file curroncol-33-00203-s001.zip › curroncol-4176517-supplementary.pdf]

Table S1 Supplementary: Multivariable analysis #1 evaluating factors associated with M1 status in the locally advanced (stage III) cohort, including age, cT, cN and grade

**Variable**	**Odds Ratio**	**95% Confidence Interval**	**P-value**
**Age**	1.02	0.99 – 1.04	0.087
**cT stage**			
1	0.52	0.05 – 5.06	0.575
2	Ref		
3	0.90	0.32 – 2.53	0.844
4	2.61	1.06 – 6.45	0.038
**cN stage**			
0	0.74	0.21 – 2.56	0.629
1	Ref		
2	0.96	0.39 – 2.40	0.936
3	2.85	1.40 – 5.80	0.004
**Grade**			
1-2	Ref		
3	0.41	0.23 – 0.74	0.003

cT: clinical tumor stage. cN: clinical nodal stage. HR: Hormone receptor. TNBC: Tripple negative breast cancer. HER2: human epidermal growth factor receptor 2.

Table S2 Supplementary: Multivariable analysis #2 evaluating factors associated with M1 status in the locally advanced (stage III) cohort, including age, overall AJCC stage and grade.

**Variable**	**Odds Ratio**	**95% Confidence Interval**	**P-value**
**Age**	1.02	0.99 – 1.04	0.099
**AJCC Overall Stage**			
IIIA	0.26	0.12 – 0.54	<0.001
IIIB	Ref		
IIIC	1.57	0.82 – 2.99	0.173
**Grade**		0.153 – 21.806	0.634
1-2	Ref		
3	0.40	0.22 – 0.72	0.002

AJCC: American Joint Committee on Cancer.
